# Genetic analysis of potential biomarkers and therapeutic targets in ferroptosis from coronary artery disease

**DOI:** 10.1111/jcmm.17239

**Published:** 2022-02-13

**Authors:** Xun Wu, Kele Qin, Chukwuemeka Daniel Iroegbu, Kun Xiang, Jun Peng, Jianjun Guo, Jinfu Yang, Chengming Fan

**Affiliations:** ^1^ Department of the Cardiovascular Surgery The Second Xiangya Hospital Central South University Changsha China; ^2^ Hunan Provincial Key Laboratory of Cardiovascular Research Central South University Changsha China; ^3^ Hunan Fangsheng Pharmaceutical Co., Ltd. Changsha China

**Keywords:** bioinformatics, cardiac, coronary artery disease, diagnostic, ferroptosis, immune microenvironment

## Abstract

Ferroptosis plays a key role in the death of cells including cardiomyocytes, and it is related to a variety of cardiac diseases. However, the role of ferroptosis‐related genes (FRGs) in coronary artery disease (CAD) is not well characterized. We downloaded CAD‐related information and FRGs from the gene expression omnibus (GEO) database and Ferroptosis Database (FerrDb) respectively. A total of 10 CAD‐related DE‐FRGs were obtained, which were closely linked to autophagy regulation and immune response. Subsequently, *CA9*, *CBS*, *CEBPG*, *HSPB1*, *SLC1A4*, *STMN1* and *TRIB3* among the 10 DE‐FRGs were identified as marker genes by LASSO and SVM‐RFE algorithms, which had tolerable diagnostic capabilities. Subsequent functional enrichment analysis showed that these marker genes may play a corresponding role in CAD by participating in the regulation of immune response, amino acid metabolism, cell cycle and multiple pathways related to the pathogenesis of CAD. Furthermore, a total of 58 drugs targeting 7 marker genes had been obtained. On the contrary, the ceRNA network revealed a complex regulatory relationship based on the marker genes. Also, CIBERSORT analysis showed that the changes in the immune microenvironment of CAD patients may be related to *CBS*, *HSPB1* and *CEBPG*. We developed a diagnostic potency and provided an insight for exploring the mechanism for CAD. Before clinical application, further research is needed to test its diagnostic value for CAD.

## 1 INTRODUCTION

Coronary artery disease (CAD) is a type of disease in which the lumen is narrowed or blocked due to atherosclerotic lesions in the coronary artery, resulting in myocardial ischaemia, oxygen deprivation and necrosis. CAD is one of the highest mortality diseases in the world, with an estimated 12 million deaths due to coronary atherosclerosis by the end of 2030, including non‐ST‐segment elevation myocardial infarction and ST segment elevation myocardial infarction.^1^ The pathogenesis of CAD is complex, and there are no obvious symptoms in the early stage, only abnormal ST‐T changes during exercise plate electrocardiogram examination. Therefore, it is an urgent need for biomarkers that can be detected in peripheral blood to facilitate the early detection of CAD.

Ferroptosis is an iron‐dependent programmed cell death mode newly discovered in 2012,^2^ with its mechanism different from apoptosis, necrosis, pyroptosis and autophagy. Ferroptosis is characterized by mitochondrial atrophy and increased mitochondrial membrane density, the accumulation of iron and lipid reactive oxygen species (L‐ROS) and the involvement of a unique set of genes.^3^, ^4^ The biochemical mechanism of iron is catalysed by the formation of lipid radicals and the depletion of glutathione (GSH) or the inactivation of lipid peroxidase 4 (GPX4).^5^ Circulating iron plays a key role in the development of iron death. The use of iron chelating agents can inhibit iron death induced by Erastin, and the expression of transferrin on the cell membrane also increases the sensitivity of cells to iron death.^2^

Ferrostatin 1 (FER‐1), a specific inhibitor of ferroptosis, significantly reduced the cardiotoxicity induced by doxorubicin (DOX) and effectively improved the survival rate in mice treated with cell death inhibition and cell death pathway‐related gene knockout, revealing that ferroptosis is an important mechanism of myocardial injury.^6^ In addition, high levels of L‐ferritin were observed in the coronary arteries of patients with coronary heart disease, indicating iron accumulation in atherosclerotic plaques.^7^ Non‐transferrin‐bound iron (NTBI) is thought to be the pathologic trigger of iron overload, and iron in NTBI is more likely to be utilized by various plaque cell types, including endothelial cells, macrophages and vascular smooth muscle cells.^8^ The use of deferoxamine can inhibit the development of atherosclerotic lesions and reduce ferroptosis of cardiomyocytes following cardiac ischaemic‐reperfusion.^9^, ^10^ Therefore, we analysed and verified the accuracy of iron death‐related genes as biomarkers of CAD and their roles in the cardiac immune microenvironment via bioinformatics analysis.

## 2 MATERIALS AND METHODS

### 2.1 Data source

In this study, the gene expression data for CAD and normal samples were obtained from the GEO database. The GSE20680 dataset embodied a total of 139 samples, including 52 normal samples and 87 CAD samples. This dataset was considered as a training set for analysis by the main body of this research. The GSE20681 dataset containing 99 normal samples and 99 CAD samples was used to verify the expression of the marker genes. Additionally, the FRGs (*n* = 259) used in this study were obtained from FerrDb, and the detailed genes were shown in Table S1.

Drug Gene Interaction Database (DGIdb) was used to predict drugs targeting marker genes. Also, the structural information of the targeted drugs of the marker gene was retrieved from the DrugBank database.

### 2.2 Differential expression analysis

We first extracted expression data of 237 FRGs (only 237 FRGs expressed in this dataset) in normal samples and CAD samples from the GSE20680 database. Subsequently, the student's *t*‐test in R was used to detect the FRGs that were differentially expressed in two different samples (Table S2). Genes with *p* < 0.05 were considered significant.

### 2.3 Functional enrichment performed in Metascape

Metascape (http://metascape.org/) was used to analyse the potential functions associated with DE‐FRGs. These analyses included Gene Ontology (GO), Reactome pathway enrichment, and Immunologic Signatures enrichment analysis (Table S3). Among them, Immunologic Signatures database used in the immunologic signatures enrichment analysis is based on the integration of the immune‐related enrichment analysis of the target gene in the published literature. In addition, Reactome is a database of articles written by experts and peer‐reviewed on various reactions and biological pathways in the human body.

### 2.4 Identification of optimal diagnostic gene biomarkers for CAD

The least absolute shrinkage and selection operator (LASSO) algorithm was applied with the glmnet package to reduce the dimensions of the data.^11^, ^12^ The DE‐FRGs between CAD patients and normal samples were retained for feature selection, and gene biomarkers for CAD were identified with the LASSO algorithms. Meanwhile, a support vector machine‐recursive feature elimination (SVM‐RFE) model was established with a SVM package, which was compared by the average misjudgement rates of their 10‐fold cross‐validations.^13^ Furthermore, optimal gene biomarkers for CAD were identified by overlapping biomarkers derived from the two algorithms. The diagnostic ability of the optimal gene biomarkers was assessed by calculating the receiver operating characteristic (ROC) curve, and measuring the area under the curve (AUC), accuracy, sensitivity and specificity. Furthermore, a logistic regression model based on seven marker genes was constructed to predict the sample types in the GSE20680 dataset using the predict function through the R package glm. Similarly, the diagnostic power of the logistic regression model was evaluated using ROC curves.

### 2.5 Single‐gene Gene Set Enrichment Analysis (GSEA) enrichment analysis

This analysis is implemented in the GSEA (V.4.1.0) package in R. To further explore the related pathways of the seven marker genes, we calculated the correlation between the marker genes and all other genes in the GSE20680 dataset. Subsequently, all genes were sorted according to their correlations from high to bottom, and these sorted genes were considered to be the gene set to be tested. Meanwhile, the KEGG signalling pathway set was invoked as a predefined set to detect its enrichment in the gene set. Specific enrichment results of each marker gene were integrated into Table S4.

### 2.6 Single‐gene Gene Set Variation Analysis (GSVA) enrichment analysis

This analysis was implemented in the GSVA (V.1.38.0) package in R. GSVA is a gene set variation analysis.^14^ In this study, we utilized the KEGG pathway set as the background gene set to perform GSVA analysis on each marker gene. Simultaneously, we applied the limma package to analyse the difference in GSVA score of the marker gene's high‐ and low‐expression group samples. The difference screening condition was |t| >2, *p* < 0.05. If t > 0, we considered the pathway to be activated in the high‐expression group, on the contrary, if t < 0, we considered the pathway to be activated in the low‐expression group. Specific enrichment results of each marker gene were integrated into Table S5.

### 2.7 Immune infiltration analysis

CIBERSORT, a method to characterize the cell composition of complex tissues from the gene expression profile.^15^ In this study, we predicted the proportion of 22 types of infiltrating immune cell types in each tissue from the GSE20680 dataset by CIBESORT software (Table S6). For each sample, the sum of all evaluated immune cell type fractions equalled 1.^16^

### 2.8 Construction of ceRNA network

The starBase database was used to predict mRNA‐miRNA interaction pairs based on the 7 marker genes. Meantime, RNA sequences of 7 marker genes were downloaded from National Center for Biotechnology Information (NCBI), and the human miRNA sequences were obtained from miRbase. The miranda software predicted the binding of mRNA‐miRNA nucleic acid, and the binding score threshold was increased to 170 (the default was 140). Then, we searched the predicted miRNA in starBase and screened miRNA‐lncRNA so that we obtained the ceRNA network of mRNA‐miRNA‐lncRNA.

### 2.9 Statistical analysis

The comparison between the two groups used the Student's t‐test. Pearson correlation analysis was used to reveal the relationship between 10 DE‐FRGs. The drawing of the Venn diagram was achieved through the Jvenn package. Cytoscape was used to visualize the ceRNA network. *p* < 0.05 was regarded as significant. All analyses were performed in R.

## 3 RESULTS

### 3.1 Identification of DE‐FRGs in the GSE20680 cohort

**FIGURE 1:**
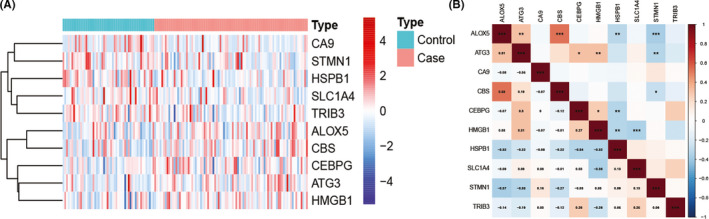
DE‐FRGs expression levels in CAD. (A) Violin plots show expression patterns of DE‐FRGs across samples. (B) The correlation of these genes. *HSPB1* had a negative correlation with *ALOX5*, *ATG3*, *CBS*, *CEBPG* and *HMGB1*. *ATG3* was positively correlated with *ALOX5*, *CBS*, *CEBPG* and *HMGB1*. Interestingly, *TRIB3* and *CA9* were not correlated with any DE‐FRGs

Ten of 237 FRGs were differentially expressed between CAD and normal samples, including 5 up‐regulated and 5 down‐regulated genes, which were identified from the GSE20680 dataset **(**Table 1). The clustering heatmap showed the expression pattern of DE‐FRGs among samples (Figure 1A). The correlation between these genes was presented in Figure 1B. *HSPB1* had a negative correlation with *ALOX5*, *CBS*, *CEBPG*, *ATG3* and *HMGB1*. *ATG3* was positively correlated with *ALOX5*, *CBS*, *CEBPG* and *HMGB1*. Interestingly, *TRIB3* and *CA9* were not correlated with any DE‐FRGs.

**TABLE 1:** Ten of 237 FRGs were differentially expressed between CAD and normal samples, including 5 up‐regulated and 5 down‐regulated genes

Gene	*p*‐Value	Expressing trend
STMN1	0.013295	DN
HSPB1	0.013299	DN
CBS	0.020652	UP
HMGB1	0.027065	UP
SLC1A4	0.027545	DN
CEBPG	0.03259	UP
TRIB3	0.034862	DN
ATG3	0.037813	UP
ALOX5	0.039414	UP
CA9	0.048775	DN

### 3.2 Functional analyses for the DE‐FRGs

**FIGURE 2:**
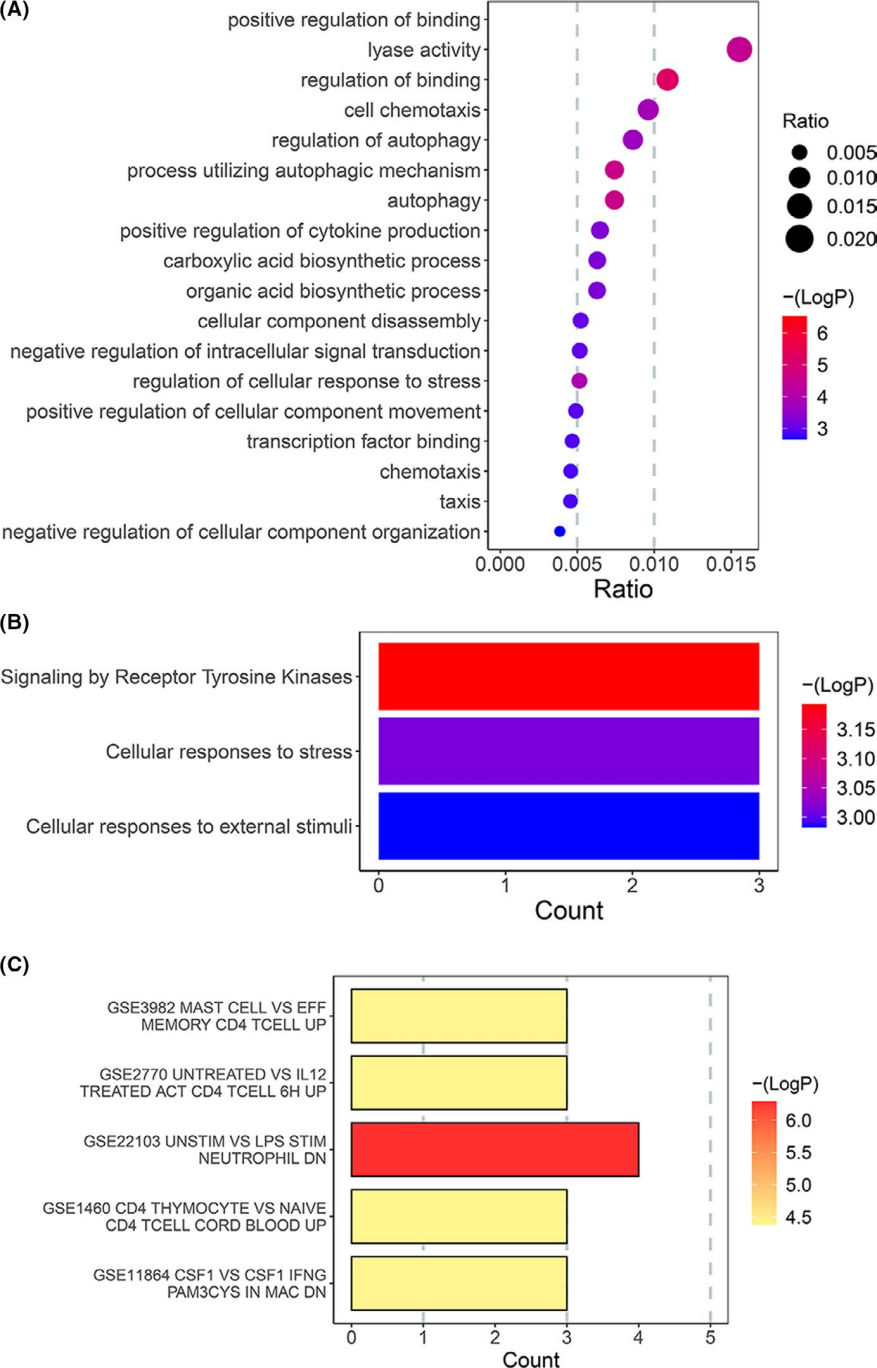
Functional analyses for the DE‐FRGs. (A) GO enrichment analyses indicated that DE‐FRGs were significantly related to the function of ‘positive regulation of binding’, ‘autophagy’ and ‘positive regulation of cytokine production’. (B) Reactome pathway analyses indicated that the cellular responses to stress, cellular responses to external stimuli, and signalling by receptor tyrosine kinases were enriched. (C) Enrichment analysis of immune characteristic gene sets

To elucidate the biological functions and pathways that were associated with the DE‐FRGs, GO enrichment and Reactome pathway analyses were perform. Consequently, GO enrichment analyses indicated that DE‐FRGs were significantly related to the function of ‘positive regulation of binding’, ‘autophagy’ and ‘positive regulation of cytokine production’ **(**Figure 2A). Reactome pathway analyses indicated that the signalling by receptor tyrosine kinases, cellular responses to stress and external stimuli were enriched (Figure 2B). Interestingly, the DE‐FRGs were also obviously enriched in many immune‐related signatures (Figure 2C). These pieces of evidence indicated that DE‐FRGs may play a role in the pathogenesis of CAD by participating in the regulation of autophagy, immune cells, cytokines and a variety of kinases.

### 3.3 7 DE‐FRGs were identified as diagnostic genes for CAD

**FIGURE 3:**
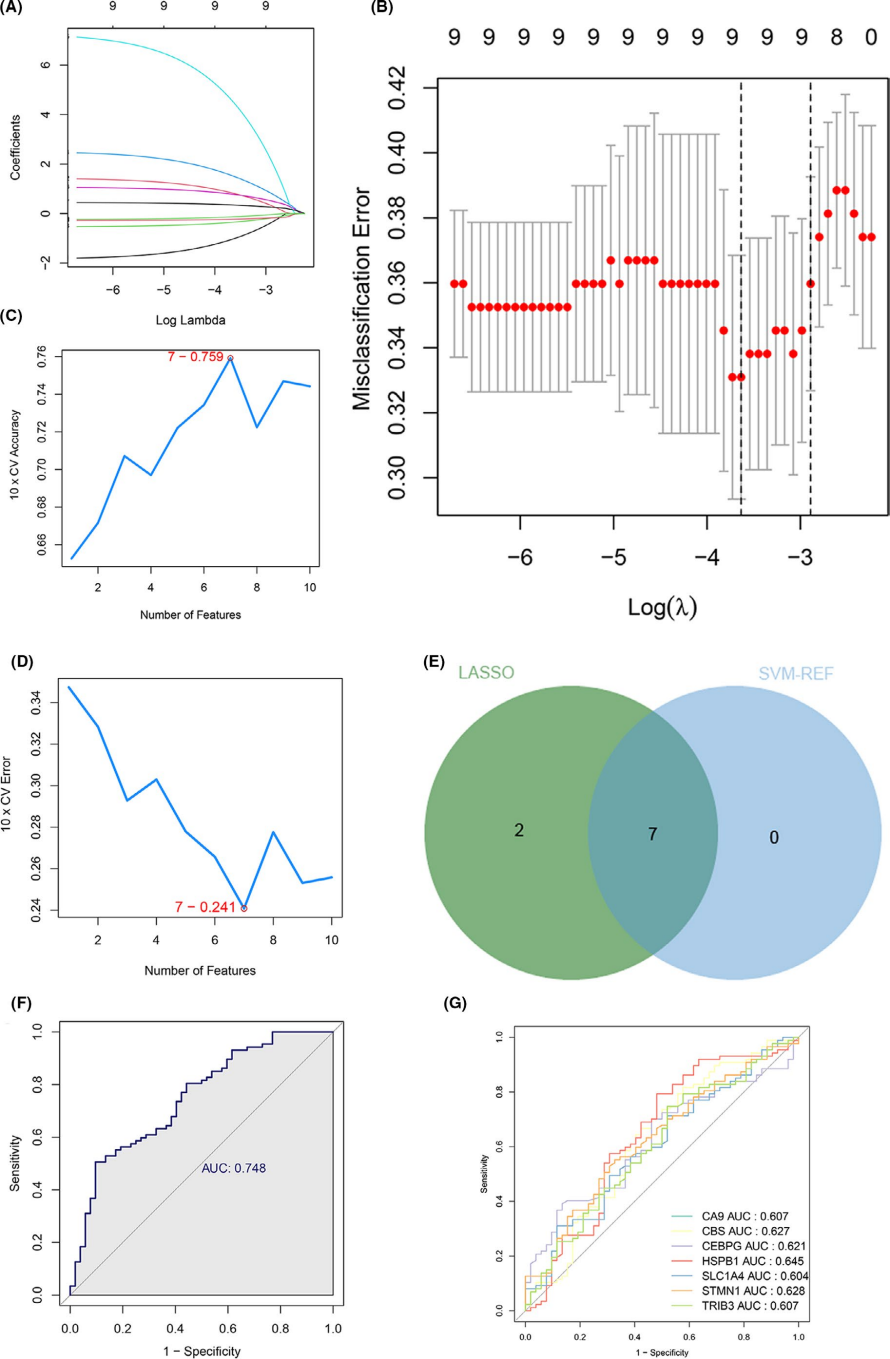
7 DE‐FGs were identified as diagnostic genes for CAD. (A and B) By LASSO logistic regression algorithm, with penalty parameter tuning conducted by 10‐fold cross‐validation, was used to select 9 CAD‐related features. (C and D) SVM‐RFE algorithm to filter the 10 DE‐FRGs to identify the optimal combination of feature genes. Finally, 7 genes (maximal accuracy =0.759, minimal RMSE =0.241) were identified as the optimal feature genes. (E) The marker genes obtained from the LASSO and SVM‐RFE models. (F) Logistic regression model to identify the AUC of disease samples. (G) ROC curves for the 7 marker genes

For considering the variation between CAD patients and healthy people, we aimed to estimate the diagnostic potential of DE‐FRGs. Next, we performed two distinct machine learning algorithms in the GSE20680 dataset, the LASSO and SVM‐RFE, to screen the significant DE‐FRGs to distinguish CAD from normal people. LASSO logistic regression algorithm, with penalty parameter tuning conducted by 10‐fold cross‐validation, was used to select 9 CAD‐related features (Figure 3A,B). We then applied the SVM‐RFE algorithm to filter the 10 DE‐FRGs to identify the optimal combination of feature genes. Finally, 7 genes (maximal accuracy =0.759, minimal RMSE =0.241) were identified as the optimal feature genes (Figure 3C,D). The marker genes obtained from the LASSO and SVM‐RFE models were intersected, and 7 marker genes (*CA9*, *CBS*, *CEBPG*, *HSPB1*, *SLC1A4*, *STMN1* and *TRIB3*) were identified for subsequent analysis (Figure 3E).

Based on the above 7 marker genes, we constructed a logistic regression model by R package glm, and the subsequent ROC curves indicated that the 7 marker gene‐based logistic regression model differentiated normal and CAD samples with AUC = 0.748 (Figure 3F). Moreover, to elucidate the ability of individual genes in distinguishing CAD from normal samples, ROC curves were generated for the 7 marker genes. As shown in Figure 3G, the AUC for all genes was greater than 0.6. The above evidence suggested that for differentiating CAD samples from normal samples, the logistic regression model provided a superior accuracy and specificity than the individual marker genes.

### 3.4 Marker genes were closely linked to a variety of CAD‐related pathways

**FIGURE 4:**
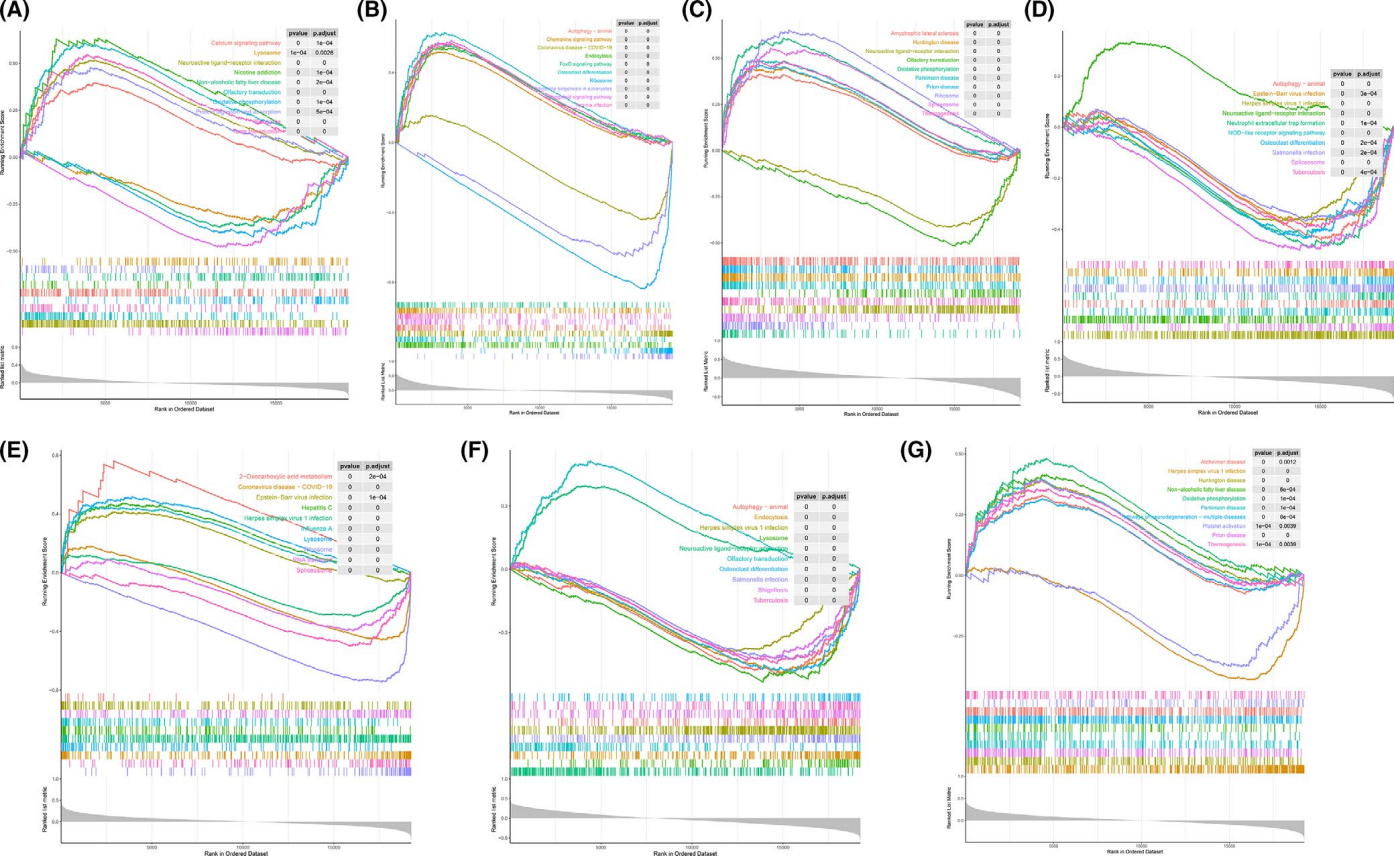
Single‐gene GSEA‐KEGG pathway analysis in CA9 (A), CBS (B), CEBPG (C), HSPB1 (D), SLC1A4 (E), STMN1(F) and TRIB3 (G)

To further explore the potential function of marker genes to distinguish diseased samples from normal samples, we conducted a single‐gene GSEA‐KEGG pathway analysis. The top10 pathways enriched for each marker gene were illustrated in Figure 4A–G. After a comprehensive analysis, we found that these genes were enriched in ribosomes, autophagy, lysosomes, cell cycle, immune response (‘Neutrophil extracellular trap formation’, ‘T‐cell receptor signalling pathway’, ‘ECM‐receptor interaction’ and ‘B‐cell receptor signalling pathway’), amino acid synthesis and metabolism (‘Caline, leucine and isoleucine degradation’, ‘Tyrosine metabolism’ and ‘Biosynthesis of amino acids’) and various disease pathways (‘Renal cell carcinoma’, ‘Hepatitis B’, ‘Coronavirus disease—COVID‐19’ and ‘Alzheimer disease’). Moreover, we found that the marker genes were also enriched in the ‘TNF signalling pathway’, ‘MAPK signalling pathway’, ‘Rap1 signalling pathway’, ‘ErbB signalling pathway’, ‘JAK‐STAT signalling pathway’, ‘PI3K‐Akt signalling pathway’, ‘TGF‐beta signalling pathway’, ‘Wnt signalling pathway’, ‘Ras signalling pathway’ and ‘mTOR signalling pathway’. Besides, we also found that *CEBPG* was closely related to the ‘Regulation of actin cytoskeleton’ (also enriched in *SLC1A4*, *STMN1* and *CBS*), ‘Vascular smooth muscle contraction’, ‘Dilated cardiomyopathy’, ‘Relaxin signalling pathway’, ‘Cardiac muscle contraction’ and ‘Hypertrophic cardiomyopathy’.

**FIGURE 5:**
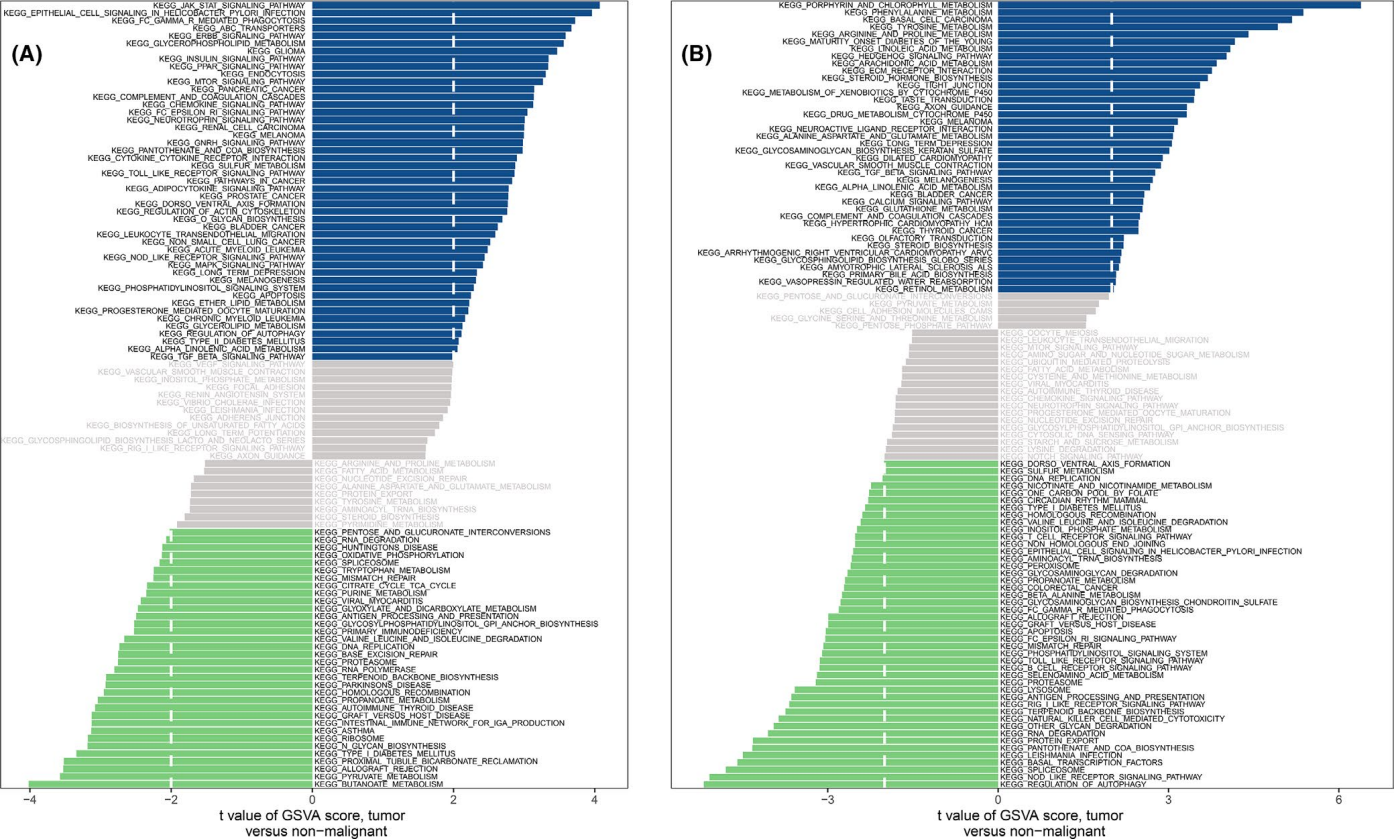
High‐ and low‐expression groups based on the expression levels of each marker gene combined with GSVA in CBS (A) and HSPB1 (B)

Then, we observed the differentially activated pathways between the high‐ and low‐expression groups based on the expression levels of each marker gene combined with GSVA. The results showed that the low expression of *CA9* in the disease may induce CDA by activating amino acid degradation, fatty acid metabolism, peroxidase and oxidative phosphorylation, while the overexpression of *CA9* activated ‘VASCULAR SMOOTH MUSCLE CONTRACTION’ and ‘ARRHYTHMOGENIC RIGHT VENTRICULAR CARDIOMYOPATHY ARVC’ **(** Figure S1A). The up‐regulation of *CEBPG* activated the amino acid metabolism/degradation and ‘CARDIAC MUSCLE CONTRACTION’ pathways (Figure S1B). Low‐expression of *SLC1A4* was only related to ‘RIBOSOME’, while the high expression of this gene activated the ‘CELL CYCLE’, amino acid metabolism (arginine and proline) and ‘REGULATION OF ACTIN CYTOSKELETON’ (Figure S1C). Many CAD‐related pathways, such as Wnt, PPAR, TGF‐β, JAK‐STAT, mTOR and ErbB signalling pathways were activated by the low expression of *STMN1*. Incredibly, the highly expressed *STMN1* was directly related to ‘CARDIAC MUSCLE CONTRACTION’ and ‘ARRHYTHMOGENIC RIGHT VENTRICULAR CARDIOMYOPATHY ARVC’ (Figure S1D). In the *TRIB3* low‐expression group, ‘HYPERTROPHIC CARDIOMYOPATHY HCM’, ‘DILATED CARDIOMYOPATHY’ and immune‐related pathways (‘CELL ADHESION MOLECULES CAMS’, ‘CHEMOKINE SIGNALLING PATHWAY’, ‘LEUKOCYTE TRANSENDOTHELIAL MIGRATION’) were enriched. The highly expressed *TRIB3* played a central role in the metabolism and degradation of amino acids (Figure S1E). It is worth noting that for *CBS*, a variety of pathways related to CAD pathogenesis were enriched in its high‐expression group, such as ‘JAK‐STAT SIGNALLING PATHWAY’, ‘ERBB SIGNALLING PATHWAY’, ‘PPAR SIGNALLING PATHWAY’, ‘MTOR SIGNALLING PATHWAY’, ‘MAPK SIGNALLING PATHWAY’ and ‘TGF BETA SIGNALLING PATHWAY’ (Figure 5A). Furthermore, *HSPB1*, whose expression was inhibited in CAD tissue, was more closely related to immune response (‘T‐CELL RECEPTOR SIGNALLING PATHWAY’, ‘B‐CELL RECEPTOR SIGNALLING PATHWAY’ and ‘NATURAL KILLER CELL‐MEDIATED CYTOTOXICITY’), ‘REGULATION OF AUTOPHAGY’ and amino acid metabolism pathway. Unreasonably, the high‐expression of *HSPB1* activated the pathways such as ‘ARACHIDONIC ACID METABOLISM’, ‘STEROID HORMONE BIOSYNTHESIS’, ‘DILATED CARDIOMYOPATHY’, ‘VASCULAR SMOOTH MUSCLE CONTRACTION’, ‘TGF BETA SIGNALLING PATHWAY’, ‘HYPERTROPHIC CARDIOMYOPATHY HCM’ and ‘ARRHYTHMOGENIC RIGHT VENTRICULAR CARDIOMYOPATHY ARVC’ that may induce CAD (Figure 5B).

### 3.5 Immune landscape analysis

**FIGURE 6:**
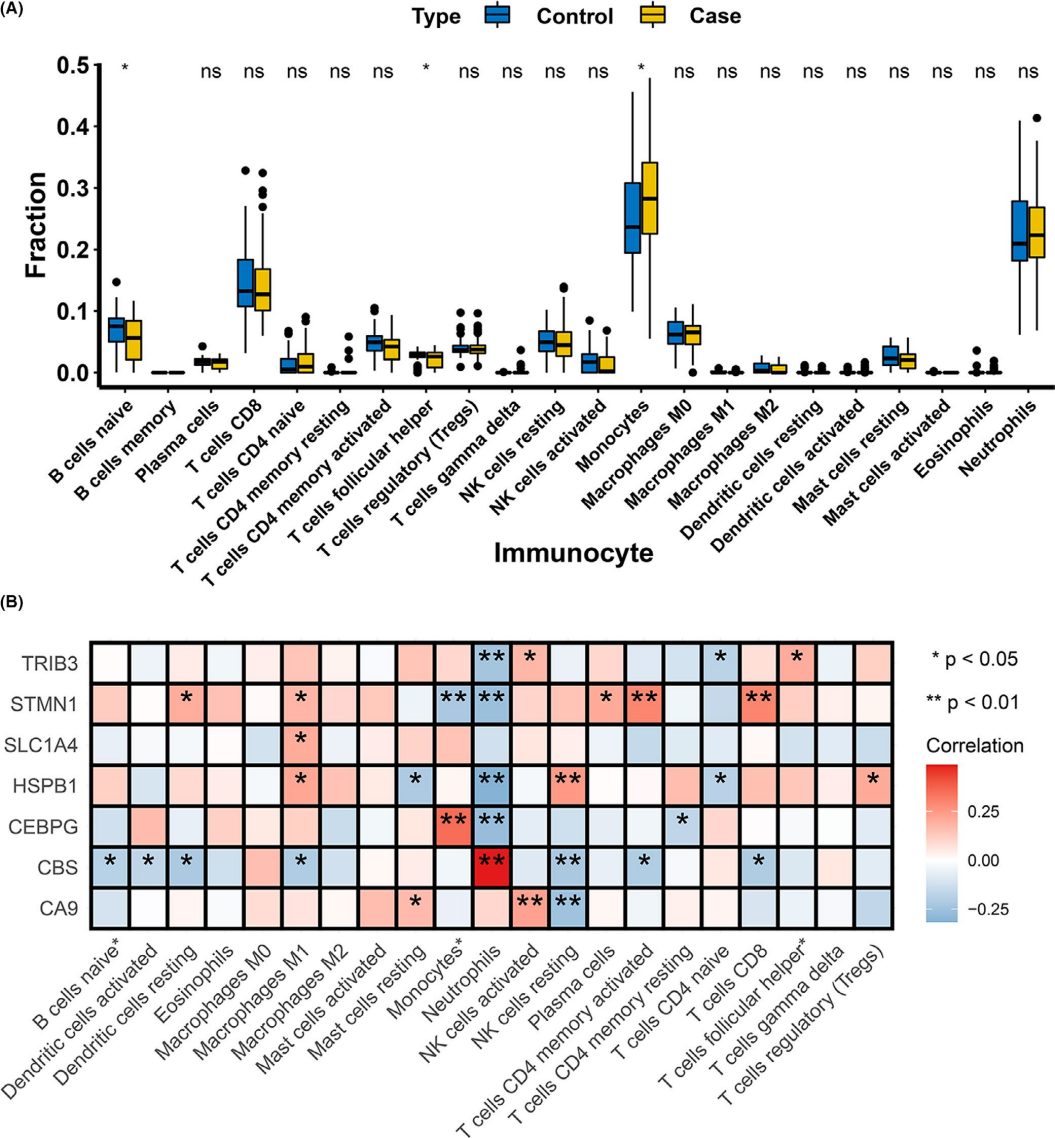
Immune landscape analysis. (A) Implemented the CIBERSORT algorithm to explore the differences in the immune microenvironment between CAD patients and normal samples. (B) Pearson correlation analysis revealed that neutrophils had strong positive and negative correlations with *CBS* and *HSPB1* respectively. *CEBPG* was positively correlated with monocytes (*p < 0.05, **p < 0.01)

The previous results indicated that the marker genes were closely related to the immune response. Meanwhile, much pieces of evidence pointed to the inseparable connection between the immune microenvironment and CAD.^17^, ^18^, ^19^ Therefore, we implemented the CIBERSORT algorithm to explore the differences in the immune microenvironment between CAD patients and normal samples. As shown in Figure 6A, the proportion of B‐cell naive in CAD samples was lower than that in normal samples, while T‐cell follicular helper and monocytes were more expressed in CAD samples. In addition, Pearson correlation analysis revealed that neutrophils had strong positive and negative correlations with *CBS* (*r* = 0.487659, *p* = 1.14E‐09) and *HSPB1* (*r* = −0.31684, *p* = 0.000145) respectively. *CEBPG* was positively correlated with monocytes (*r* = 0.35092, *p* = 2.28E‐05) (Figure 6B; Table S7). These pieces of evidence indicated that changes in the immune microenvironment of CAD patients may be linked to *CBS*, *HSPB1* and *CEBPG*.

### 3.6 Prediction of marker gene‐targeted drugs

**FIGURE 7:**
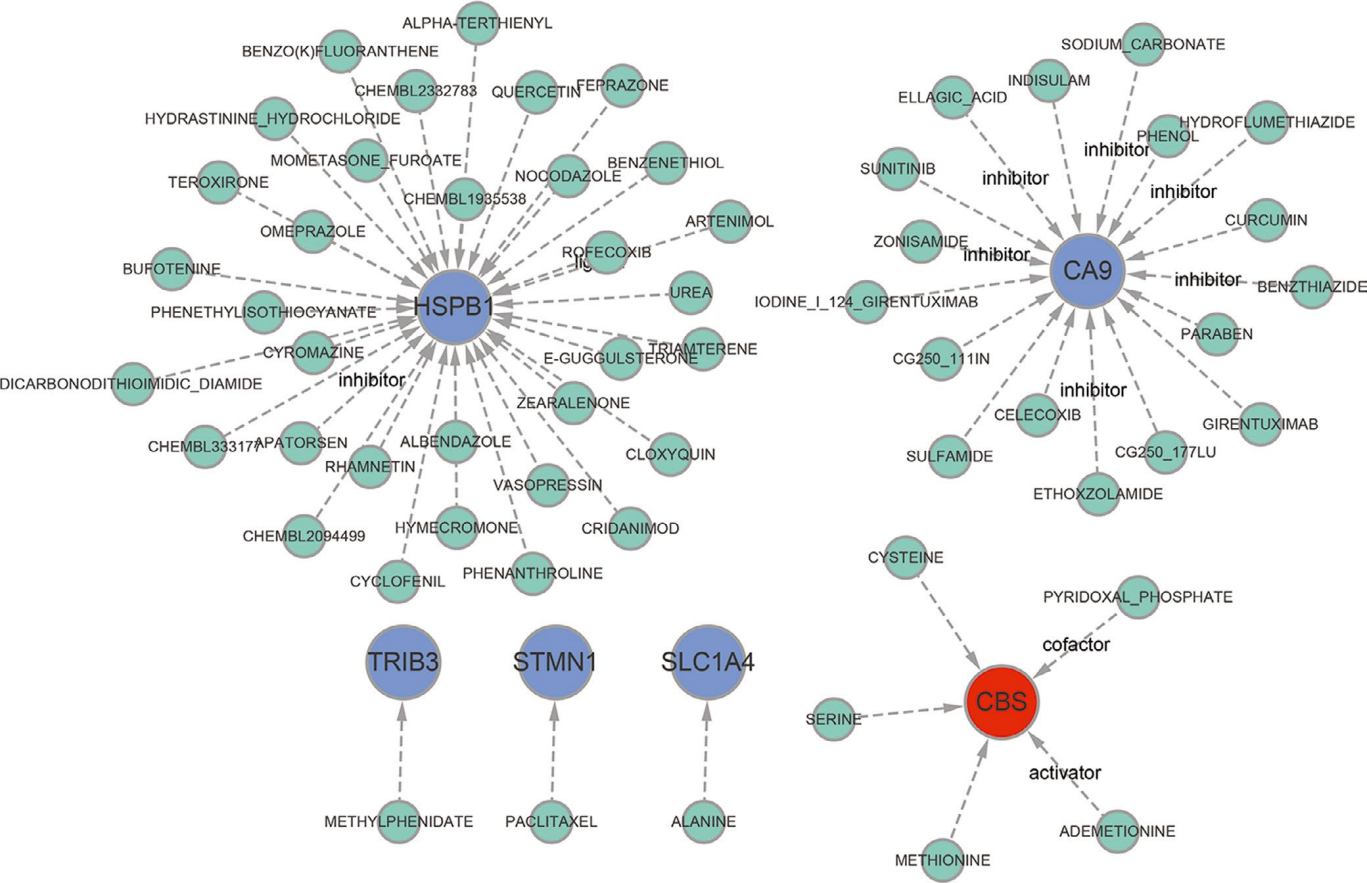
Prediction of marker gene‐targeted drugs. The drugs may target marker genes through the DGIdb database and the interaction relationship between the two

We further revealed the drugs that may target marker genes through the DGIdb database and analysed the interaction relationship between the two parameters were set to default values; (Table S8). The results visualized by Cytoscape software were shown in (Figure 7). We had queried 58 drugs targeting marker genes, including 17 for *CA9*, 5 for *CBS*, 33 for *HSPB1*, 1 for *SLC1A4*, *STMN1* targeted 1 drug and *TRIB3* targeted 1 drug. Unfortunately, we did not forecast *CEBPG*'s targeted drugs. Moreover, we also used the DrugBank database to retrieve the structural formulas of the 58 drugs mentioned above. A total of 36 drug structures were retrieved. A total of 11 drug structures were retrieved from 17 *CA9* targeted drugs. Among them, benzthiazide, ellagic acid, ethoxzolamide, hydroflumethiazide, sodium carbonate and zonisamide were known inhibitors of *CA9*. The structural formulas of *CBS*'s five targeted drugs had been retrieved, and ademetionine and pyridoxal phosphate as its activator and cofactor respectively. 17 of 33 *HSPB1* targeted drugs were displayed, of which apatorsen and artenimol were inhibitors and ligands of the gene respectively. The structural information of the targeted drugs of *SLC1A4*, *STMN1* and *TRIB3* was all revealed. The detailed results were illustrated in (Figure S2).

### 3.7 A ceRNA networks based on marker genes

**FIGURE 8:**
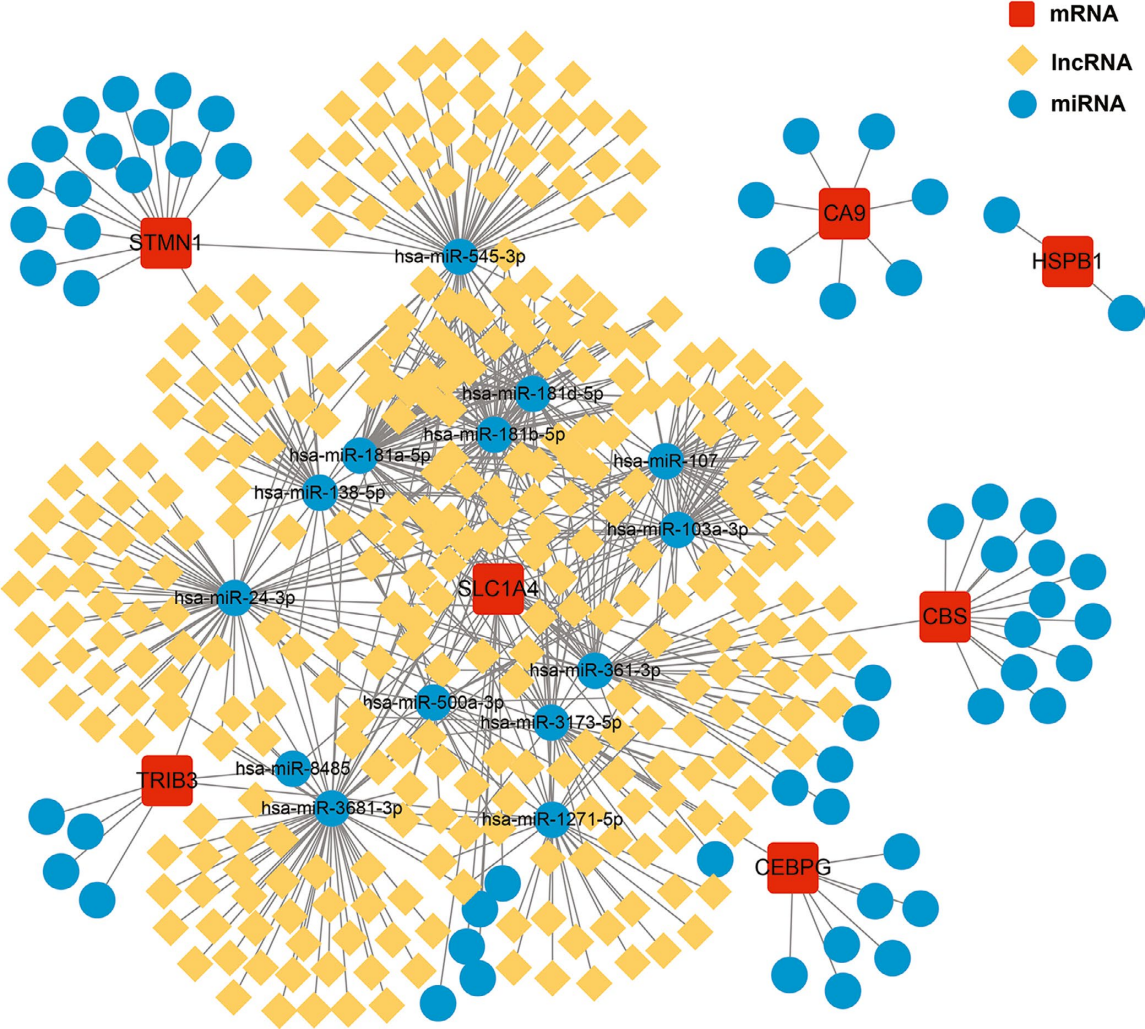
A ceRNA networks based on marker genes. The network included 408 nodes (7 marker genes, 74 miRNAs and 327 lncRNAs) and 688 edges

Next, we constructed a ceRNA network based on 7 marker genes through starBase and miranda databases. The network included 408 nodes (7 marker genes, 74 miRNAs and 327 lncRNAs) and 688 edges (Figure 8). In detail, we found that a total of 170 lncRNAs could competitively bind *hsa*‐*miR*‐*103a*‐*3p*, *hsa*‐*miR*‐*500a*‐*3p*, *hsa*‐*miR*‐*181b*‐*5p*, *hsa*‐*miR*‐*3681*‐*3p*, *hsa*‐*miR*‐*181d*‐*5p*, *hsa*‐*miR*‐*181a*‐*5p* and *hsa*‐*miR*‐*107* regulated *SLC1A4*. Among them, *hsa*‐*miR*‐*103a*‐*3p* and *hsa*‐*miR*‐*107* were shared 48 lncRNAs. In addition, 57 shared lncRNAs could target *hsa*‐*miR*‐*181a*‐*5p*, *hsa*‐*miR*‐*181b*‐*5p* and *hsa*‐*miR*‐*181d*‐*5p* respectively. For *TRIB3*, we found that 28 lncRNAs could regulate the expression of *TRIB3* through competitive binding with *hsa*‐*miR*‐*1271*‐*5p*. Meanwhile, *hsa*‐*miR*‐*1271*‐*5p* could be bound to 61 lncRNAs to exert its regulatory role in this gene. Among them, lncRNA *EBLN3P* could simultaneously target *hsa*‐*miR*‐*1271*‐*5p* and *hsa*‐*miR*‐*1271*‐*5p*. In the ceRNA network of *STMN1*, there were 40 and 56 lncRNAs that could combine with *hsa*‐*miR*‐*138*‐*5p* and *hsa*‐*miR*‐*545*‐*3p* to regulate the gene. Among these, lncRNAs, *LINC00294*, *NEAT1* and *LINC00665* were shared lncRNAs. A total of 34 lncRNAs could be competitively bound with *hsa*‐*miR*‐*3173*‐*5p* to affect the expression of *CEBPG*. The expression of *CBS* could be regulated by a competitive collection of 40 lncRNAs and *hsa*‐*miR*‐*361*‐*3p*. Specific details of the ceRNA network were shown in Table S9.

### 3.8 Expression of the marker gene in the validation set

**FIGURE 9:**
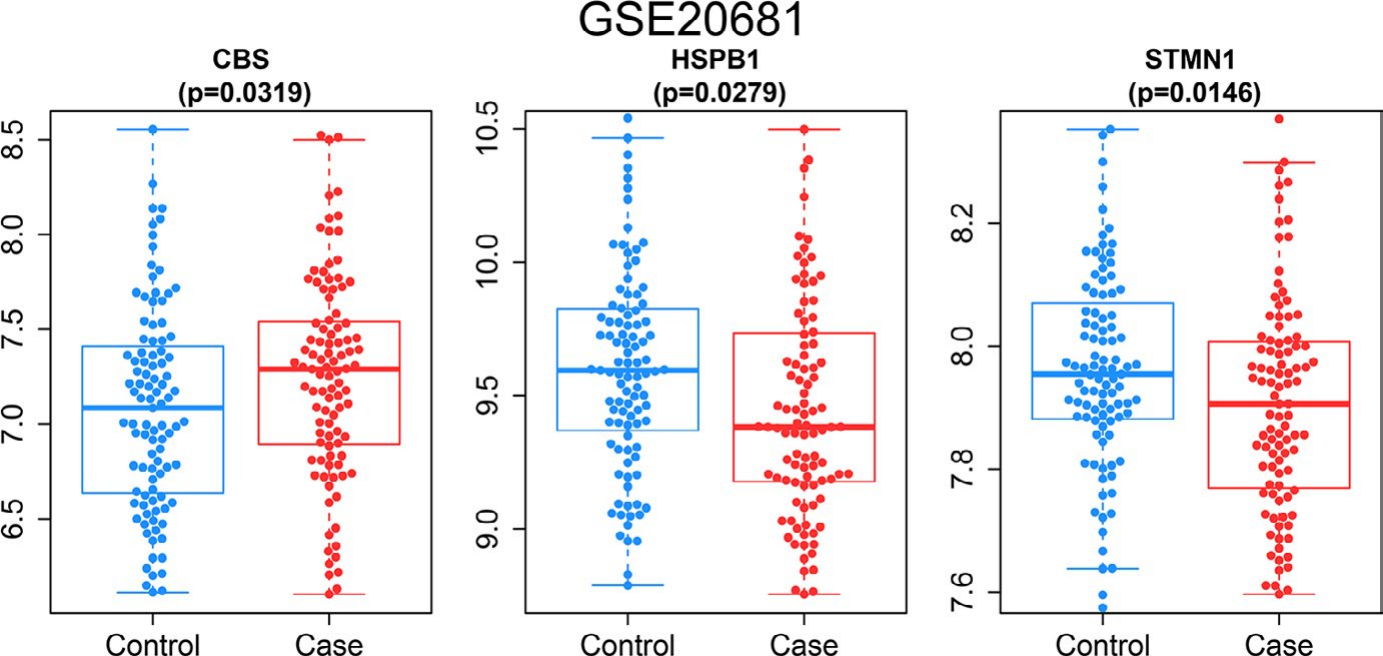
Expression of the marker gene in the validation set. The expression of marker genes in the GSE20681 dataset

Finally, we also verified the expression of marker genes in the GSE20681 dataset. We discovered that the expression trends of *CBS*, *HSPB1* and *STMN1* were consistent with the GSE20680 dataset. Among them, the expression of *CBS* (*p* = 0.0319) in CAD patients was greater than of normal samples, while the *HSPB1* (*p* = 0.0279) and *STMN1* (*p* = 0.0146) were lower in CAD samples (Figure 9).

## 4 DISCUSSION

Coronary atherosclerosis is a complex, persistent and progressive inflammatory disease characterized by remodelling of the coronary arteries that deliver oxygen and nutrition to the cardiac tissue. It has a variety of clinical manifestations, ranging from asymptomatic to stable angina, acute coronary syndrome (ACS), sudden cardiac death (SCD) or heart failure (HF). Due to the nature of the disease, most patients may suffer from coronary atherosclerosis for many years, even decades.^20^, ^21^

Atherosclerosis is the basic pathogenesis of CAD. Abnormal apoptosis of vascular endothelial cells (VECs), macrophages or vascular smooth muscle cells (VSMCs) is a common feature of atherosclerosis, which can lead to the formation of atherosclerotic plaques or plaque instability.^22^ Endothelial cell repair after injury is associated with multiple genes, some of which are also involved in the maintenance of normal cardiac function and the formation of aortic aneurysms.^23^, ^24^, ^25^ Autophagy is also involved in the process of coronary atherosclerosis, but its specific role is controversial. Some studies suggest that it is a protective factor against atherosclerosis, and that autophagy is inhibited in patients with cardiovascular disease, featured by the decrease of LC3 and Atg5 genes.^26^ However, other contrary findings suggest that autophagy worsens coronary atherosclerosis.^27^ It is worth noting that the relationship between coronary atherosclerosis and ferroptosis has not yet been reported. Thus it is important for the selection of gene chip data, two kinds of disease by gene expression data analysis, enrichment of single‐gene analysis, enrichment of bioinformatics methods, such as, finding a common point, to analyse genetic differences in CAD and normal ferroptosis, explore the molecular pathogenesis in CAD ferroptosis. In the present article, a large number of gene chips most suitable for this research was selected and the use of multiple samples of genes as well as a large number of microarray data makes the experimental results more reliable and reduces the error rate, thus providing a valuable clinical reference for the treatment and prevention of CAD.

A total of 7 differential genes related to ferroptosis were screened in this study, including *CA9*, *CBS*, *CEBPG*, *HSPB1*, *SLC1A4*, *HSPB1* and *TRIB3*. The AUC values represented by the area under the ROC curve of the 7 genes are all greater than 0.6, indicating that these 7 genes have certain accuracy and specificity for distinguishing coronary artery disease samples from normal samples. Among them, the AUC values of *CEPBG*, *HSPB1* and *CBS* rank the top 3. *HSPB1* is considered to be a molecular companion involved in regulating the composition of the cytoskeleton.^28^ After phosphorylation, the oligomer of *HSPB1* is dissociated into monomer, which then acts as an inhibitor of apoptosis and an inducer of autophagy,^29^, ^30^ The GSAV analysis of *HSPB1* also confirmed that the autophagy regulatory pathway was activated in the low‐expression group. *HSPB1* is a key regulator of ferroptosis in cancer cells, and *HSPB1* is a negative regulator of ferroptosis by reducing iron‐mediated production of lipid reactive oxygen species.^31^ Other studies have shown that *HSPB1* plays an important role in the progression of liver cancer and ferroptosis, and that *HSPB1* may be regulated by transcription factor ATF3.^32^ Studies have found that protease degradation is more abundant in atherosclerosis than in normal arteries.^33^ Therefore, the decrease of *HSPB1* in serum of patients with coronary artery disease may be caused by over‐activation of protease, or excessive consumption of *HSPB1* caused by anti‐ferroptosis in the body in the process of atherosclerosis plaque formation, which needs further study. CBS‐catalysed transsulfuration converts homocysteine to cysteine. Cysteine γ lyase converts homocysteine to cysteine.^34^ Cysteine synthesis (or cysteine uptake by cells) is the rate‐limiting step in the production of glutathione, a ubiquitous antioxidant. Studies have shown that structural activation of NRF2/CBS signalling confers Erastin‐induced ferroptosis resistance, and that *CBS* overexpression increases intracellular cysteine, supplemental to the decrease in intracellular cysteine caused by Erastin inhibition of the System XC^−^ system.^35^ *CBS* enzyme deficiency can lead to hyperhomocysteinemia, leading to premature development of cardiac and cerebrovascular disease.^36^ H_2_S is also produced from the transsulfuration of homocysteine into cysteine catalysed by *CBS*. H_2_S has been proved to play an important role in the process of anti‐atherosclerosis.^37^ Thus, *CBS* plays a role in both atherosclerosis and ferroptosis. Our study found that *CBS* is highly expressed in the CAD group, which may be a protective mechanism of the body against the disease and control the continued development of the disease.

Immune cells maintain the homeostasis of the heart and all kinds of immune cells that reside or penetrate into the heart tissue play an important role in the process of injury repair.^17^ The immune cells identified in the heart include macrophages, monocytes, neutrophils, dendritic cells (DC), T and B cells, eosinophils and mast cells, which also play an important role in maintaining heart function.^38^ Our analysis shows that T‐cell helper and monocytes are highly expressed in the CAD group, while B‐cell naive is lower than the normal group. Circulating monocytes and resident vascular macrophages are the first white blood cells known as early atherosclerotic plaques.^39^ Local inflammation is caused by damaged endothelial cells that release monocyte chemotactic protein‐1 (also known as C‐C motifchemotactic factor ligand 2 (CCl2)). Monocyte chemokine protein‐1 interacts with chemokine receptor 2 and chemokine receptor 4 expressed on circulating monocytes to recruit them to the lesions.^40^, ^41^ Migrating monocytes differentiate into macrophages, leading to inflammation and plaque development.^42^ Another study shows that T‐cell follicular helper promotes atherosclerosis, and its consumption reduces atherosclerosis.^43^ These studies have been confirmed in our analysis. CBS was positively correlated with neutrophils among different iron death‐related genes. *CBS* is a key enzyme for H_2_S production in vivo, and H2S can stimulate neutrophils adhesion and neutrophils tissue infiltration. These effects are associated with the up‐regulation of various adhesion receptors and proinflammatory mediators.^44^, ^45^ Other studies have found that H_2_S inhibits neutrophil tissue infiltration.^46^, ^47^ Whether the positive correlation between *CBS* and neutrophils in CAD patients is mediated by H_2_S remains to be verified. HSPB1 has been shown to have a cell protective effect.^48^ *HSPB1* is involved in a number of important physiological functions, including inhibition of cytokine expression and inhibition of neutrophil infiltration.^49^ My study found that *HSPB1* was negatively correlated with neutrophils, and the expression of HSPB1 was decreased in CAD patients. Therefore, *HSPB1* may be a potential target for improving cardiac immune microenvironment in CAD patients.

Finally, we analysed the marker gene for gene‐targeted drugs and the ceRNA network. Among the five *CBS* target drugs retrieved, ademetionine was confirmed to be an allosteric activator of *CBS*.^50^ The drug has been used in mentally related diseases and liver diseases,^51^, ^52^ and its application in cardiovascular diseases has not been reported. Pyridoxal phosphate acts as a cofactor for *CBS* in the body, and its deficiency will lead to the loss of *CBS* activity.^53^ The combination of the antisense oligonucleotide Apatorsen of *HSPB1* mRNA and a variety of anti‐cancer drugs enhances the effect of anti‐cancer drugs.^54^, ^55^ As a predictive targeted drug of *HSPB1*, cloxyquin has been proved to play a cardioprotective effect by regulating autophagy,^56^ but the study of whether *HSPB1*plays a role in this process has not been clarified. Non‐coding RNA plays an important role in the development of atherosclerosis, miR‐18a‐5p, miR‐27a‐3p, miR199a‐3p, miR‐223‐3p and miR‐652‐3p abundance and atherosclerosis Closely related to cardiovascular‐related reocclusion.^57^ Whether our predicted gene‐targeted drugs and non‐coding RNA can play a role is unclear, and the specific pathways need to be further studied. Therefore, the selected drugs and non‐coding RNA can be prospectively studied.

*CA9*, *CBS*, *CEBPG*, *HSPB1*, *SLC1A4*, *HSPB1* and *TRIB3* are genes that we have screened for iron death in coronary heart disease samples. Among them, we focus on the two genes*—CBS* and *HSPB1*. These two genes are not only related to iron death. In addition to being closely related, it may also be involved in the regulation of the cardiac immune microenvironment of CAD patients. Although gene expression may not be directly equivalent to protein expression, the biomarkers in this study should be regarded as genes, not proteins, but the significance of its research cannot be denied. We will continue to pay attention to these genes to deepen our understanding of the pathogenesis and treatment of coronary heart disease.

## CONFLICT OF INTEREST

The authors declare that they have no competing interests.

## AUTHOR CONTRIBUTIONS

**Xun Wu:** Data curation (equal); Formal analysis (equal); Methodology (equal); Software (equal); Writing – original draft (equal); Writing – review & editing (equal). **Kele Qin:** Methodology (equal); Writing – review & editing (equal). **Chukwuemeka Daniel Iroegbu:** Data curation (equal); Writing – review & editing (equal). **Kun Xiang:** Methodology (equal); Writing – review & editing (equal). **Jun Peng:** Investigation (equal); Writing – review & editing (equal). **Jianjun Guo:** Investigation (equal); Writing – review & editing (equal). **Jinfu Yang:** Investigation (equal); Writing – review & editing (equal). **Chengming Fan:** Investigation (equal); Project administration (equal); Supervision (equal); Writing – review & editing (equal).

## Supporting information

Figure S1

Figure S2

Table S1

Table S2

Table S3

Table S4

Table S5

Table S6

Table S7

Table S8

Table S9

## Data Availability

The datasets generated and/or analysed during the current study are available in the following locations: the gene expression data for CAD and normal samples were obtained from the GEO database: ttp://www.ncbi.nlm.nih.gov/geo/query/acc.cgi?acc=GSE20680 and http://www.ncbi.nlm.nih.gov/geo/query/acc.cgi?acc=GSE20681. Additionally, the FRGs (*n* = 259) used in this study were obtained from FerrDb: http://www.zhounan.org/ferrdb/. Drug Gene Interaction Database (http://biogps.org/plugin/1136/dgidb‐the‐drug‐gene‐interaction‐database/) was used to predict drugs targeting marker genes. Lastly, the structural information of the targeted drugs of the marker gene was retrieved from the DrugBank database (https://go.drugbank.com/).
