# Supplementary figures and images for: Genetic analysis of potential biomarkers and therapeutic targets in ferroptosis from coronary artery disease
Source: J Cell Mol Med. 2022 Feb 13;26(8):2177–90. doi: 10.1111/jcmm.17239 (PMC8995456; doi:10.1111/jcmm.17239)

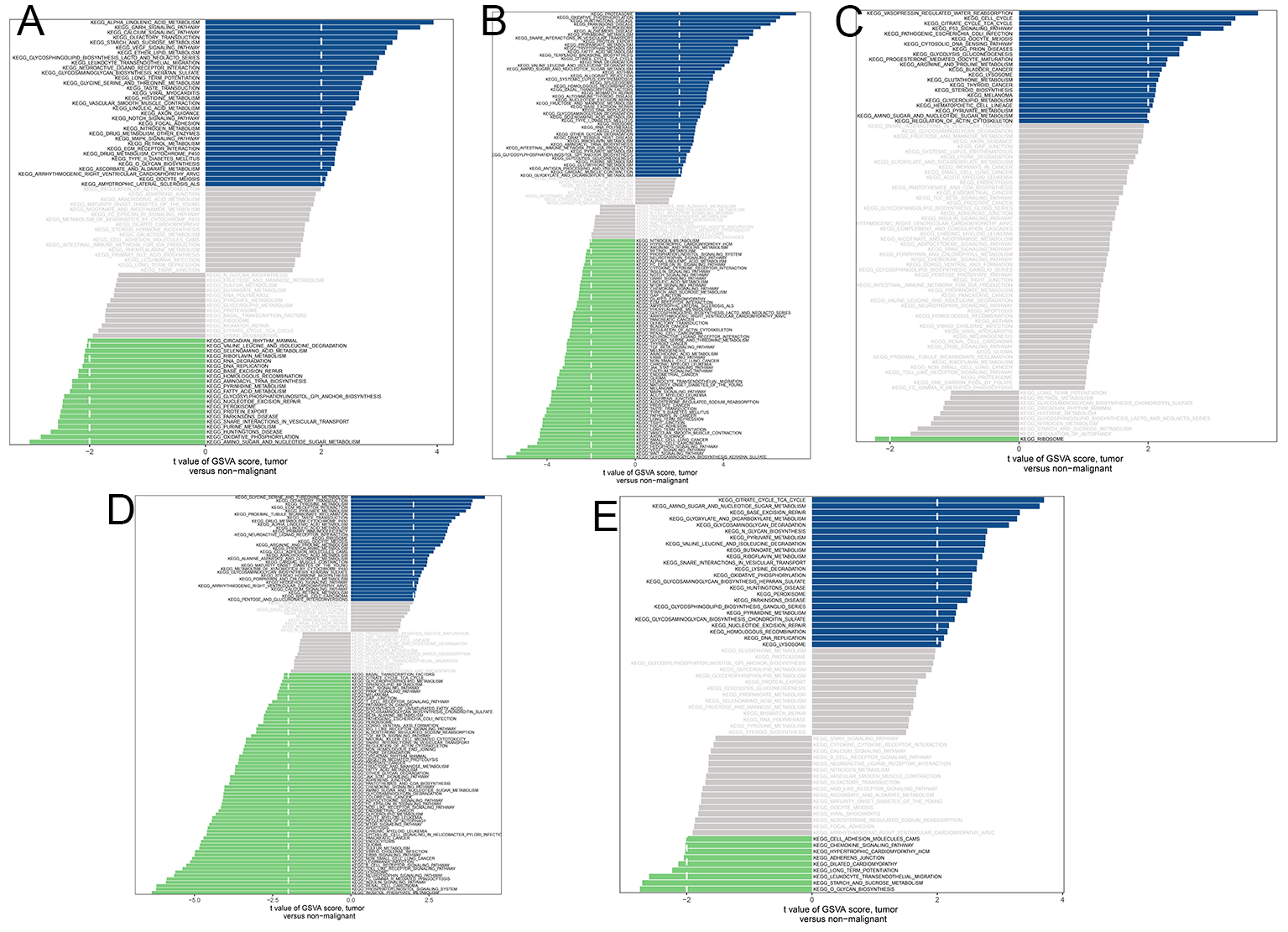

Supplement: Supplementary file 1 — Figure S1 [file JCMM-26-2177-s006.tif]

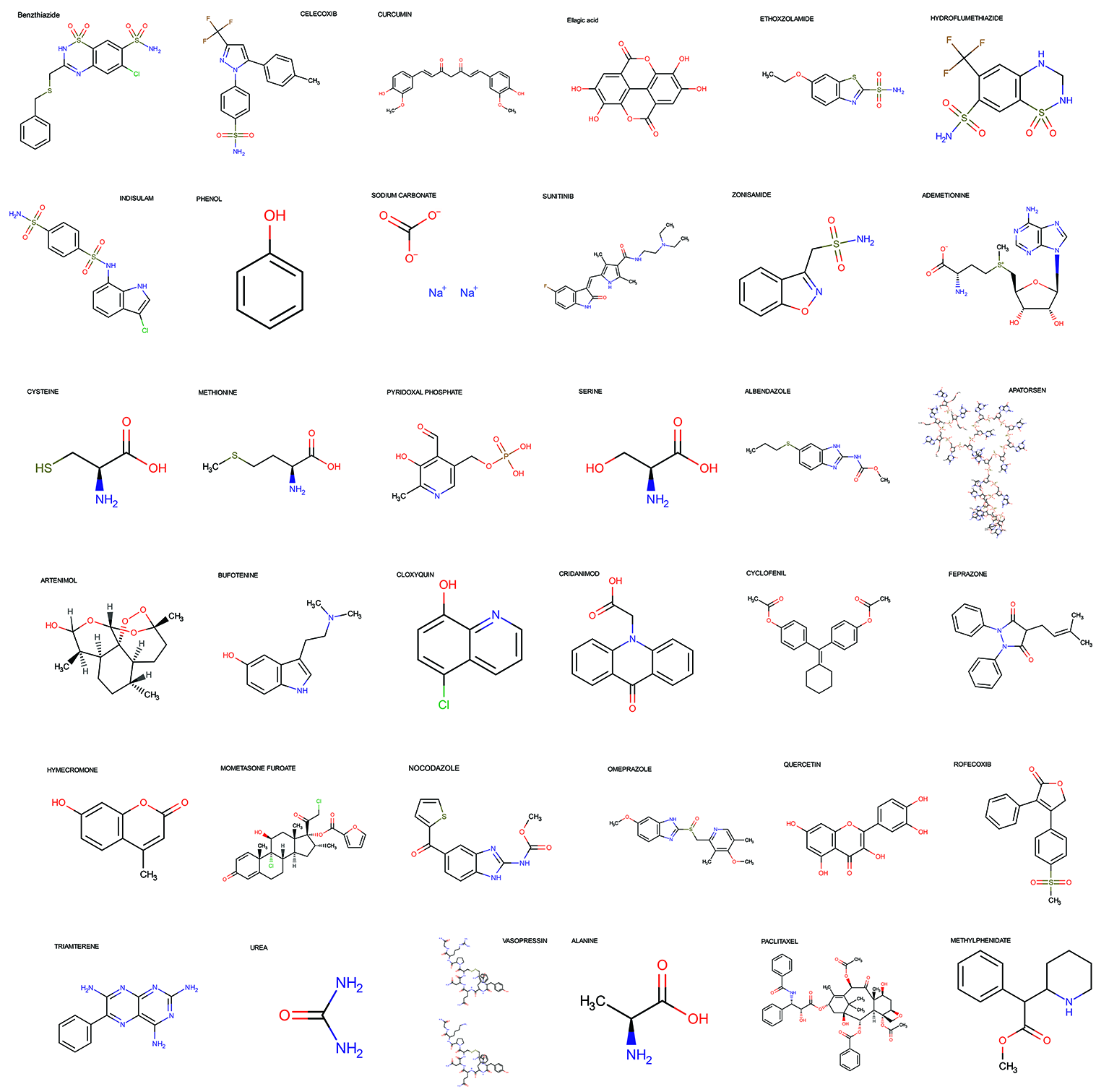

Supplement: Supplementary file 2 — Figure S2 [file JCMM-26-2177-s005.tif]
